# Radiomics-based ultrasound models for thyroid nodule differentiation in Hashimoto’s thyroiditis

**DOI:** 10.3389/fendo.2023.1267886

**Published:** 2023-10-23

**Authors:** Mengyuan Fang, Mengjie Lei, Xuexue Chen, Hong Cao, Xingxing Duan, Hongxia Yuan, Lili Guo

**Affiliations:** ^1^ Department of Ultrasound, Changsha Hospital for Maternal & Child Health Care Affiliated to Hunan Normal University, Changsha, China; ^2^ State Key Laboratory of Oncology in South China, Collaborative Innovation Center for Cancer Medicine, Sun Yat-sen University Cancer Center, Guangzhou, China; ^3^ Institute of Clinical Medicine, The First Affiliated Hospital of University of South, Hengyang, Hunan, China; ^4^ Department of Ultrasound, The People’s Hospital of Guangxi Zhuang Autonomous Region, Nanning, China

**Keywords:** thyroid nodules, Hashimoto’s thyroiditis, ultrasound, radiomics, diagnosis

## Abstract

**Background:**

Previous models for differentiating benign and malignant thyroid nodules(TN) have predominantly focused on the characteristics of the nodules themselves, without considering the specific features of the thyroid gland(TG) in patients with Hashimoto’s thyroiditis(HT). In this study, we analyzed the clinical and ultrasound radiomics(USR) features of TN in patients with HT and constructed a model for differentiating benign and malignant nodules specifically in this population.

**Methods:**

We retrospectively collected clinical and ultrasound data from 227 patients with TN and concomitant HT(161 for training, 66 for testing). Two experienced sonographers delineated the TG and TN regions, and USR features were extracted using Python. Lasso regression and logistic analysis were employed to select relevant USR features and clinical data to construct the model for differentiating benign and malignant TN. The performance of the model was evaluated using area under the curve(AUC), calibration curves, and decision curve analysis(DCA).

**Results:**

A total of 1,162 USR features were extracted from TN and the TG in the 227 patients with HT. Lasso regression identified 14 features, which were used to construct the TN score, TG score, and TN+TG score. Univariate analysis identified six clinical predictors: TI-RADS, echoic type, aspect ratio, boundary, calcification, and thyroid function. Multivariable analysis revealed that incorporating USR scores improved the performance of the model for differentiating benign and malignant TN in patients with HT. Specifically, the TN+TG score resulted in the highest increase in AUC(from 0.83 to 0.94) in the clinical prediction model. Calibration curves and DCA demonstrated higher accuracy and net benefit for the TN+TG+clinical model.

**Conclusion:**

USR features of both the TG and TN can be utilized for differentiating benign and malignant TN in patients with HT. These findings highlight the importance of considering the entire TG in the evaluation of TN in HT patients, providing valuable insights for clinical decision-making in this population.

## Summary

Previous models for differentiating benign and malignant thyroid nodules(TN) have predominantly focused on the characteristics of the nodules themselves, without considering the specific features of the thyroid gland(TG) in patients with Hashimoto’s thyroiditis(HT). it is worth further investigating whether US features of TN and TG play an important role in the benign-malignant discrimination of TN in patients with HT. In this study, clinical and US data were retrospectively collected from 227 patients with HT accompanied by TN. A total of 1,162 USR features were extracted from TN and the TG in the 227 patients with HT. Lasso regression identified 14 features, which were used to construct the TN score, TG score, and TN+TG score. Multivariable analysis revealed that incorporating USR scores improved the performance of the model for differentiating benign and malignant TN in patients with HT. Specifically, the TN+TG score resulted in the highest increase in AUC(from 0.83 to 0.94) in the clinical prediction model. Calibration curves and DCA demonstrated higher accuracy and net benefit for the TN+TG+clinical model. In conclusion, USR features of both the TG and TN can be utilized for differentiating benign and malignant TN in patients with HT.

## 1 Introduction

Hashimoto’s thyroiditis (HT), an autoimmune disease, is the most common cause of hypothyroidism, characterized by diffuse lymphocytic infiltration and progressive autoimmune reactions leading to chronic inflammation and thyroid dysfunction (1, 2). On the other hand, thyroid cancer (TC) is the most common malignancy of the endocrine system, with rapidly increasing incidence rates globally, ranging from 4.5% to 6.6% per year (3, 4). Thyroid nodules (TN) are a common presentation of TC, but TN are not always malignant (5). Differentiating between benign and malignant TN is crucial for detecting TC, which has significant implications for guiding treatment decisions, improving patients’ quality of life, and optimizing healthcare resources (6, 7). Numerous etiological and epidemiological studies have indicated a higher coexistence rate of HT and TC, estimated at approximately 23% (ranging from 10% to 58%) (8). However, the current assessment systems used to distinguish between benign and malignant conditions often overlook the impact of HT on TN, which could lead to a lower detection rate of TC in HT patients.

Ultrasound (US) is widely used in the evaluation of TN because it is a non-invasive and radiation-free imaging technique that provides detailed structural information (9, 10). The American College of Radiology Thyroid Imaging Reporting and Data System (ACR TI-RADS) is currently the most commonly used tool in clinical practice for risk stratification of TN. This system encompasses five ultrasound features, including composition, echogenicity, shape, margins, and echogenic foci (11). It has been reported that ACR TI-RADS has a sensitivity of approximately 88% and specificity of around 49%. However, some malignant TNs exhibit benign features in ultrasound images, such as smooth margins and absence of calcification. Therefore, the evaluation value of ACR TI-RADS for these types of TNs is limited (12). To improve the accuracy of US diagnosis of TN, researchers are constantly exploring new image features and classification algorithms (13). For example, Zhao et al. proposed a local and global feature disentanglement network to classify the benign and malignant nature of thyroid nodules, achieving an accuracy of 89.33% (14). Recently, radiomics based on US image analysis has shown superior performance compared to other conventional methods (15). Radiomics can automatically extract a large number of quantitative image features from medical images, which are often difficult to identify by the naked eye (16, 17). Radiomics can provide complementary information to image features and, in combination with clinical information and US image features, improve model performance (18–20). Zheng et al., for instance, demonstrated the application of ultrasound radiomics (USR) to build a predictive model for better predicting the status of axillary lymph node metastasis in early-stage breast cancer patients prior to surgery (18).

HT and TN may be associated in certain cases. The chronic inflammation caused by HT can result in thyroid tissue damage and progressive structural changes, which may contribute to the formation of nodules (21). US imaging of HT presents with several unique features, including abnormal echogenicity patterns, abnormal blood flow signals, and diffuse changes (22). Previous studies on US features for benign-malignant discrimination of TN have primarily focused on the nodules themselves, while overlooking the US features of the thyroid gland (TG) which may indicate the differences between benign and malignant nodules (23–26). Jin et al. also reported that predictive models based on US features of TC and TG could effectively predict central lymph node metastasis (27). Therefore, it is worth further investigating whether US features of TN and TG play an important role in the benign-malignant discrimination of TN in patients with HT.

In this study, clinical and US data were retrospectively collected from 227 patients with HT accompanied by TN. By outlining the target areas and extracting US features of the TG and TN, we constructed a specific diagnostic model for TN benign-malignant discrimination, taking into account the patients’ clinical information.

## 2 Method

### 2.1 Patient selection

The study was conducted in accordance with the Declaration of Helsinki (as revised in 2013). From January 2012 to December 2022, we retrospectively collected 5,478 patients with TN from Changsha Hospital for Maternal & Child Health Care Affiliated to Hunan Normal University and People’s Hospital of Guangxi Zhuang Autonomous Region. The inclusion criteria are as follows: (1) TN Patients have HT. (2)All patients have undergone thyroid surgery and have tissue pathology results. (3) The diagnosis of HT and the benign or malignant nature of TN were confirmed by post-operative pathological examination. (4)ACR TI-RADS score≥4. The exclusion criteria are as follows: (1) Patients with two or more TN. (2) Lacking complete clinical data and high-quality US images. (3) Lacking pathological data for the diagnosis of TN and HT. Finally, there were 227 patients enrolled in this study.

### 2.2 Data collection

All included patients in this study had their clinical data collected, including preoperative basic clinical information, conventional ultrasound results, thyroid function indicators, and other serological markers. The basic clinical information comprised age, gender, BMI, tumor size (long diameter), and location. The conventional ultrasound results were assessed by experienced sonographers, and the obtained features included echoic type (iso/hyper/hypo/marked hypo echoic), margin (well/ill defined), calcification (NO/macro/micro calcification), vascularity (NO/low/median/high). Thyroid function indicators encompassed total triiodothyronine (TT3), free triiodothyronine (FT3), total tetraiodothyronine (TT4), free tetraiodothyronine (FT4), thyroid stimulating hormone (TSH), parathyroid hormone (PTH), anti-thyroid peroxidase (anti-TPO), thyroid globulin, anti-thyroid globulin (anti-TG) and calcitonin. Other serological markers primarily reflected inflammation and nutritional status, such as neutrophil count, lymphocyte count, platelet count, calcium levels, lactate dehydrogenase, albumin, and others.

### 2.3 Segmentation and feature extraction of US

In this study, preoperative ultrasound data in DICOM format were collected from patients. After excluding low-quality data, the high-quality ultrasound data were imported into ITK-SNAP software (Version 3.8). Segmentation of the regions of interest (ROIs) was performed using a double-blind method, with two experienced ultrasound specialists independently delineating the ROIs. The delineated target areas were compared by the two ultrasound specialists, and any discrepancies in the regions were adjusted. In cases of disagreement, a third physician provided confirmation. The ROIs delineation included two parts: TN and TG. The delineated target areas were saved in NIFF format. Finally, radiomics data were extracted using the Python package pyradiomics (V1.3.0), and a total of 1,162 USR features were extracted from the thyroid (531 from TN and 531 from TG).

### 2.4 USR feature selection and model establishment

The ROIs from the TG and TN were analyzed together. To identify the most relevant and significant features, we employed statistical methods such as independent t-test and least absolute shrinkage and selection operator (LASSO) regression. These methods helped us select a subset of features that had the strongest correlation with the target variable, and we calculated USR scores using regression techniques. Besides, logistic regression analysis was used to conduct univariate analysis on clinical and serum markers, and markers significantly associated with malignant nodule were included in the multivariate analysis. We combined the USR scores with clinically significant information, thyroid function indicators, and serum markers to perform a comprehensive multivariable analysis and establish multiple predictive models for malignant nodule.

### 2.5 Statistical analysis

All statistical analyses were performed using R software (Version 4.1.3). Continuous variables were reported as medians and interquartile ranges (IQRs), and categorical variables as frequencies and percentages. The Wilcoxon signed-rank test was used in two sets of related samples. Logistic regression analysis was used to build the lymph node prediction model and calculate the odds ratios (ORs) with relative 95% confidence intervals (95%CI) to determine the relevance of all potential predictors. In logistic regression analysis, univariate analysis was first conducted to screen for statistically significant predictive factors, and then statistically significant predictors were included in the multivariable model. In the ROC curve, the area under the curve (AUC) was used to evaluate the differences between different models. Thousand bootstrap resamples were used to internal validation of novel diagnostic models. Decision curve analysis (DCA) was performed to determine the net benefit associated with the models (28). The discrimination and DCA were corrected for overfitting using leave-one-out cross-validation. All tests were two-tailed and p<0.05 was considered statistically significant.

## 3 Results

**Figure 1:**
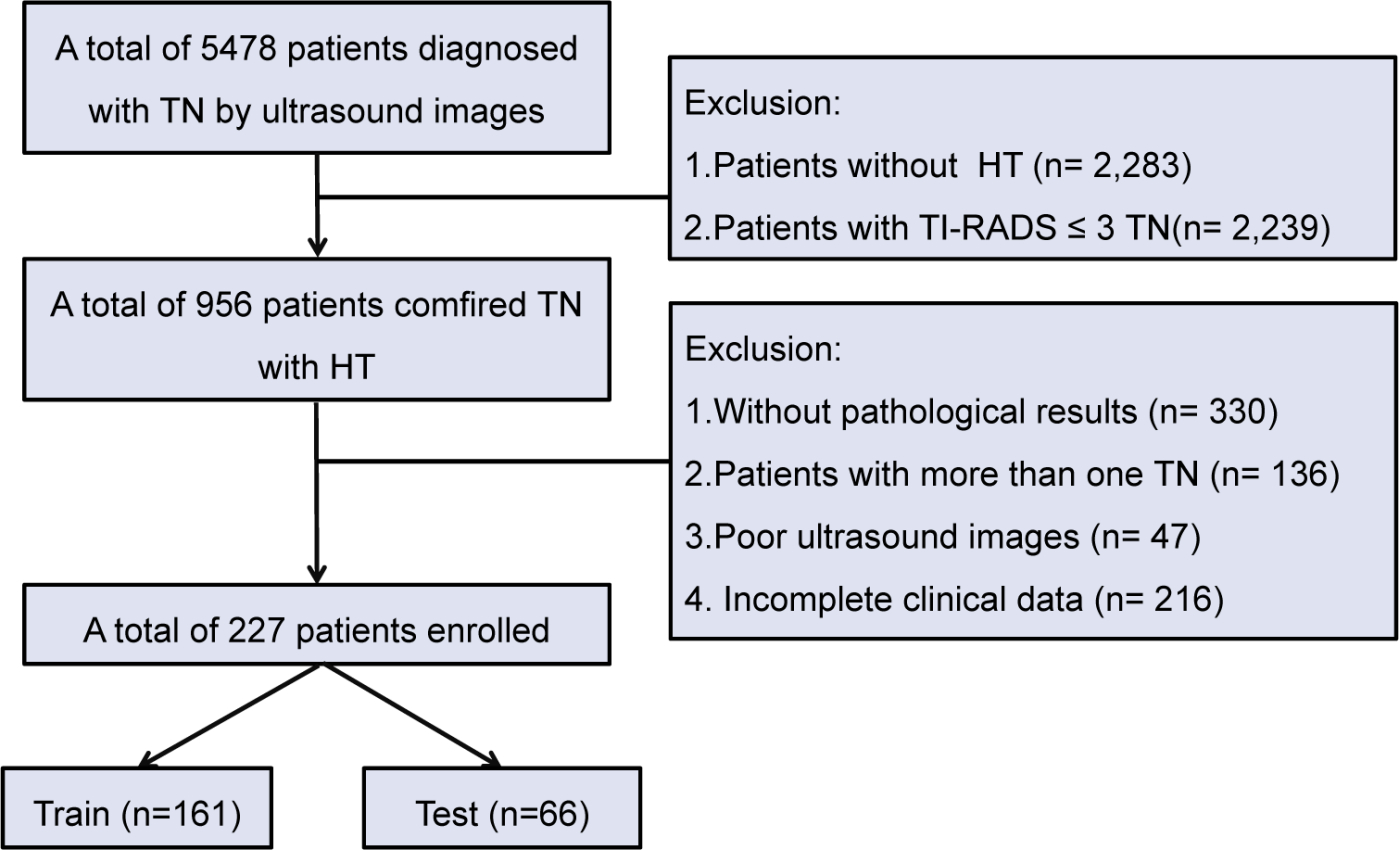
Flowchart of patient selection for TN patients with HT. TN, thyroid nodule; HT, Hashimoto’s thyroiditis.

In this study, a total of 5,478 patients with TN who underwent US examination were reviewed. Patients without HT and those with a TI-RADS score less than 3 were excluded, resulting in 956 patients with HT and TN. Further screening based on pathological results, presence of multiple nodules, ultrasound image quality, and completeness of clinical data excluded 729 patients. Finally, there was a sample size of 227 patients for inclusion including 161 patients for training and 66 patients for testing (**Figure 1**).

**Figure 2:**
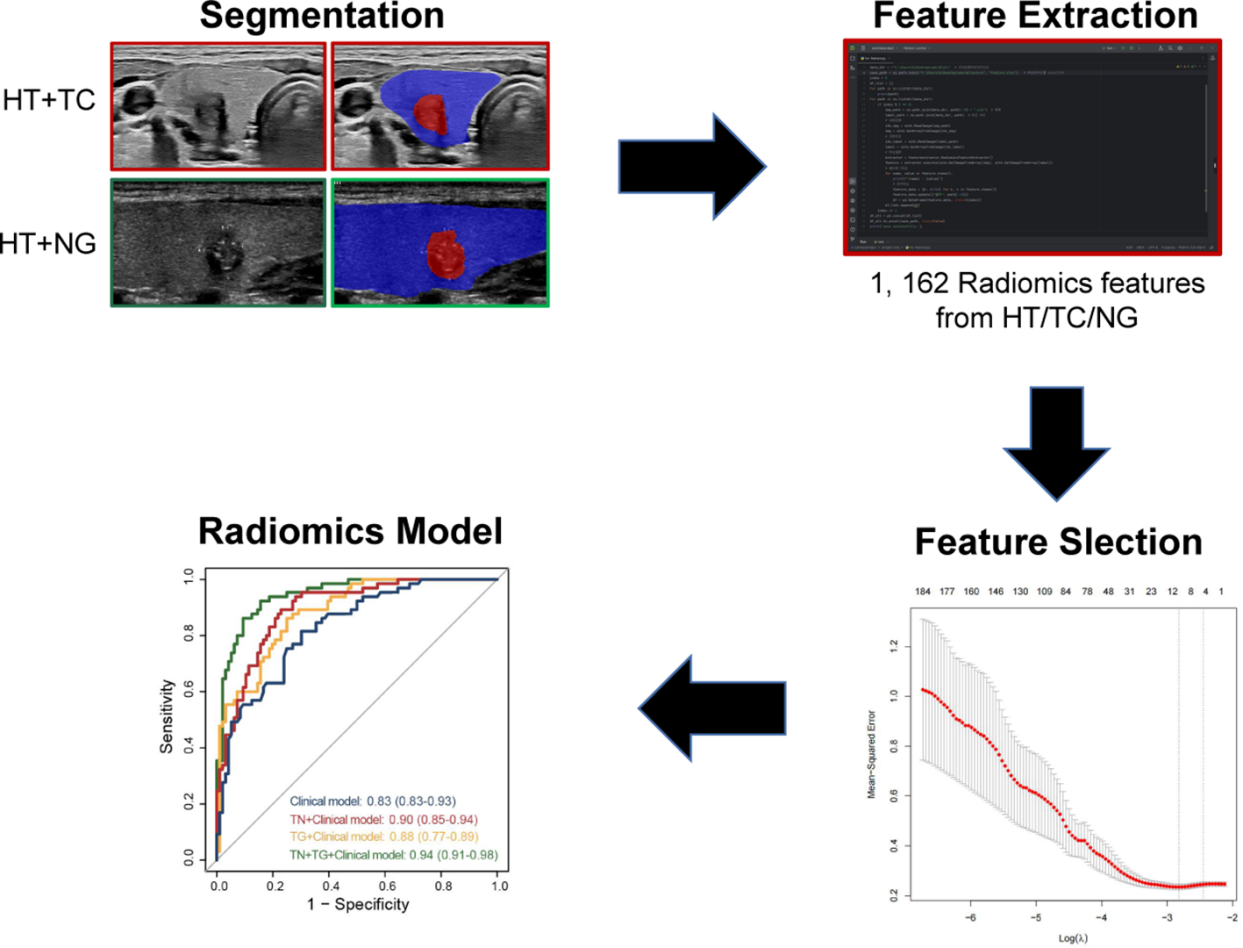
Flowchart of development of radiomics model for TN patients with HT. TN, thyroid nodule; HT, Hashimoto’s thyroiditis.

As shown in **Figure 2**, we delineated the target regions of TN (highlighted in red) and the TG (highlighted in blue) on US images for the 227 patients. A total of 1,162 USR features were extracted from both the ROIs of TN and the TG using Python. By applying LASSO regression, we ultimately identified 14 USR features (4 from TG and 9 from TN) for distinguishing benign and malignant TN. Based on these 14 USR features, we use logistics analysis to construct the TN+TG score, TN score, and TG score, respectively.

The baseline characteristics of the training and testing groups demonstrate good comparability (**Table 1**). Both groups exhibit significantly higher median levels of anti-TPO (>35 ng/mL) and anti-TG (>115 IU/mL) compared to normal levels. In both the training and validation groups, patients with TR4 and TR5 thyroid nodules each constitute around half of the total enrolled population. More than 60% of patients present with hypoechoic TN with indistinct borders. Over 50% of patients have an aspect ratio >1 and show microcalcifications in the TN. Around one-third of patients in both groups exhibit symptoms of either hyperthyroidism or hypothyroidism.

**Table 1:** Baseline characteristics.

Characteristics	Training (n=161)	Test (n=66)	p-value
Age (y), median (IQR)	38.00 (33.00, 46.00)	36.00 (31.25, 45.75)	0.751
Gender (%)			0.123
Male	20 (12.4)	3 (4.5)	
Female	141 (87.6)	63 (95.5)	
TI-RADS level (%)			0.781
TR 4	82 (50.9)	34 (51.5)	
TR 5	79 (49.1)	32 (48.5)	
Echoic type (%)			0.877
Iso/hyper echoic	34 (21.1)	14 (21.2)	
hypo echoic	111 (68.9)	42 (63.6)	
marked hypo echoic	16 (9.9)	10 (15.2)	
Aspect ratio (%)			0.690
≤1	70 (43.5)	30 (45.5)	
>1	91 (56.5)	36 (54.5)	
Boundary (%)			
clear	52 (32.3)	24 (36.4)	
unclear	109 (67.7)	42 (63.6)	
Margin (%)			0.804
well-defined	90 (55.9)	35 (53.0)	
ill-defined	71 (44.1)	31 (47.0)	
Calcification (%)			0.541
NO	31 (19.3)	15 (22.7)	
macro calcification	49 (30.4)	18 (27.3)	
micro calcification	81 (50.3)	33 (50.0)	
Vascularization (%)			0.323
NO	16 (9.9)	3 (4.5)	
low/median	98 (60.9)	42 (63.6)	
high	47 (29.2)	21 (31.8)	
Thyroid function (%)			0.443
normal	112 (69.6)	49 (74.2)	
Hyper/hypo-thyroidism	49 (30.4)	17 (25.8)	
Thyroid function index			
TSH, uIU/mL	1.97 (1.33, 3.36)	1.97 (1.53, 3.65)	0.62
PTH, pg/mL	35.79 (26.03, 45.44)	35.54 (28.05, 47.02)	0.928
anti-TPO, U/mL	72.40 (47.30, 230.00)	75.35 (38.62, 270.00)	0.962
Thyroid globulin, ng/mL	3.98 (1.07, 30.30)	5.48 (1.40, 34.42)	0.508
anti-TG, IU/mL	238.50 (49.98, 471.00)	198.50 (51.95, 434.17)	0.594
calcitonin, pg/mL	7.00 (4.13, 7.59)	7.00 (4.60, 7.48)	0.469
FT3, pmol/L	4.63 (4.29, 5.05)	4.72 (4.20, 5.27)	0.620
FT4, pmol/L	16.15 (14.69, 18.02)	16.15 (15.00, 18.90)	0.468
TT3, nmol/L	1.68 (1.48, 1.86)	1.76 (1.54, 1.98)	0.181
TT4, nmol/L	99.90 (86.58, 111.25)	99.90 (87.60, 117.00)	0.555
USR score, median (IQR)			
TG	0.42 (0.29, 0.50)	0.44 (0.29, 0.55)	0.413
TN	0.36 (0.20, 0.55)	0.36 (0.21, 0.53)	0.881
TN+TG	0.33 (0.09, 0.66)	0.38 (0.09, 0.66)	0.784

IQR, inter quartile range; TSH, thyroid stimulating hormone; PTH, parathyroid hormone; anti-TPO, anti-thyroid peroxidase; anti-TG, anti-thyroid globulin; FT3, free triiodothyronine; FT4, free tetraiodothyronine; TT3, total triiodothyronine; TT4, total tetraiodothyronine; USR, ultrasound radiomics; TG, thyroid gland; TN, thyroid nodule.

In training group, there were 96 benign nodules and 65 malignant nodules, while in testing group, there were 42 benign nodules and 65 malignant nodules (**Table 2**). In univariate analysis, we identified 6 predictive factors associated with TN malignancy in the training group: TI-RADS, echoic type, aspect ratio, boundary, calcification, and thyroid function (**Supplementary Table 1**). However, in the testing group, the correlations between boundary, calcification, and thyroid function with TN malignancy did not reach statistical significance. Both in the training and testing groups, the USR scores, including TN+TG score, TN score, and TG score, demonstrated significant statistical differences between benign and malignant TN.

**Table 2:** Predictors for TN status in the training and the test datasets.

Characteristics	Training			Test		
	Benign (n=96)	Malignant (n=65)	p value	Benign (n=42)	Malignant (n=24)	p value
Age (y), median (IQR)	36.0 (32.8, 45.0)	38.0 (33.0, 47.0)	0.833	36.50 (32.25, 45.75)	36.00 (30.50, 42.25)	0.607
Gender (%)			1.000			0.615
Male	12 (12.5)	8 (12.3)		1 (2.4)	2 (8.3)	
Female	84 (87.5)	57 (87.7)		41 (97.6)	22 (91.7)	
TI-RADS level (%)			<0.001			<0.001
TR 4	82 (50.9)	72 (75.0)		30 (71.4)	4 (16.7)	
TR 5	79 (49.1)	24 (25.0)		12 (28.6)	20 (83.3)	
Echoic type (%)			<0.001			<0.001
iso/hyper echoic	30 (31.2)	4 (6.2)		13 (31.0)	1 (4.2)	
hypo echoic	64 (66.7)	47 (72.3)		28 (66.7)	14 (58.3)	
marked hypo echoic	2 (2.1)	14 (21.5)		1 (2.4)	9 (37.5)	
Aspect ratio (%)			<0.001			0.023
≤1	56 (58.3)	14 (21.5)		24 (57.1)	6 (25.0)	
>1	40 (41.7)	51 (78.5)		18 (42.9)	18 (75.0)	
Boundary (%)			<0.001			0.086
clear	42 (43.8)	10 (15.4)		19 (45.2)	5 (20.8)	
unclear	54 (56.2)	55 (84.6)		23 (54.8)	19 (79.2)	
Margin (%)			0.706			0.363
well-defined	52 (54.2)	38 (58.5)		20 (47.6)	15 (62.5)	
ill-defined	44 (45.8)	27 (41.5)		22 (52.4)	9 (37.5)	
Calcification (%)			<0.001			0.305
NO	28 (29.2)	3 (4.6)		12 (28.6)	3 (12.5)	
macro calcification	27 (28.1)	22 (33.8)		10 (23.8)	8 (33.3)	
micro calcification	41 (42.7)	40 (61.5)		20 (47.6)	13 (54.2)	
Vascularization (%)			0.402			0.305
NO	12 (12.5)	4 (6.2)		1 (2.4)	2 (8.3)	
low/median	56 (58.3)	42 (64.6)		27 (64.3)	15 (62.5)	
High	28 (29.2)	19 (29.2)		14 (33.3)	7 (29.2)	
Thyroid function (%)			0.001			0.052
normal	77 (80.2)	35 (53.8)		35 (83.3)	14 (58.3)	
Hyper/hypo-thyroidism	19 (19.8)	30 (46.2)		7 (16.7)	10 (41.7)	
Thyroid function index, median (IQR)						
TSH, uIU/mL	2.08 (1.29, 3.69)	1.79 (1.36, 2.74)	0.190	2.42 (1.46, 4.03)	1.80 (1.59, 2.66)	0.372
PTH,	35.79 (26.31, 46.81)	35.79 (25.99, 43.30)	0.466	35.44 (28.19, 48.98)	35.54 (26.77, 45.68)	0.636
TPO,	72.40 (45.02, 258.50)	72.40 (50.80, 213.00)	0.796	75.35 (37.30, 282.25)	92.70 (53.85, 228.75)	0.957
Thyroid globulin, ng/mL	4.76 (1.16, 24.15)	3.98 (0.74, 32.70)	0.654	8.87 (1.40, 37.40)	3.98 (1.47, 25.50)	0.734
anti-TG, IU/mL	223.00 (44.97, 366.50)	278.00 (146.00, 783.00)	0.207	155.00 (51.95, 312.10)	220.00 (152.85, 649.70)	0.098
calcitonin, pg/mL	7.00 (4.11, 8.35)	6.16 (4.17, 7.00)	0.358	7.00 (4.57, 8.15)	6.58 (4.88, 7.02)	0.260
FT3, nmol/L	4.61 (4.27, 5.10)	4.67 (4.32, 4.99)	0.634	4.76 (4.18, 5.44)	4.72 (4.20, 5.05)	0.926
FT4, nmol/L	16.15 (14.60, 18.05)	16.15 (14.97, 17.90)	0.749	16.50 (14.90, 19.60)	15.95 (15.23, 18.00)	0.673
TT3, nmol/L	1.68 (1.47, 1.88)	1.68 (1.58, 1.84)	0.760	1.77 (1.46, 1.98)	1.75 (1.68, 1.94)	0.645
TT4, nmol/L	99.90 (84.50, 110.50)	99.90 (90.40, 114.70)	0.219	96.90 (86.70, 116.00)	99.90 (92.42, 122.25)	0.384
USR score, median (IQR)						
TG	0.35 (0.27, 0.48)	0.47 (0.38, 0.58)	<0.001	0.39 (0.26, 0.49)	0.50 (0.39, 0.61)	0.017
TN	0.28 (0.15, 0.40)	0.55 (0.36, 0.78)	<0.001	0.30 (0.16, 0.39)	0.58 (0.43, 0.78)	<0.001
TN+TG	0.15 (0.03, 0.36)	0.69 (0.50, 0.95)	<0.001	0.18 (0.04, 0.41)	0.70 (0.52, 0.92)	<0.001

IQR, inter quartile range; TSH, thyroid stimulating hormone; PTH, parathyroid hormone; TPO, thyroid peroxidase; anti-TG, anti-thyroid globulin; FT3, free hypothyroidism; FT4, free neurotoxin; TT3, total hypothyroidism; TT4, total neurotoxin; USR, ultrasound radiomics; TG, thyroid gland; TN, thyroid nodule.

**Figure 3:**
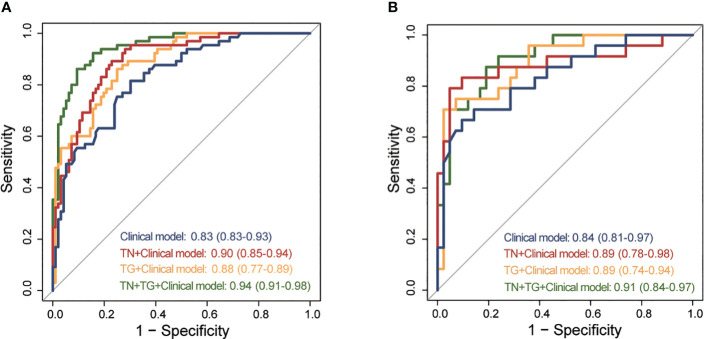
ROC of different predictive models for predicting TC in training and testing group. **(A)** ROC of different predictive models in training group. **(B)** ROC of different predictive models in testing group. ROC, receiver operating curves; TC, thyroid cancer; TN, thyroid nodule; TG, thyroid gland.

We constructed four models for distinguishing benign and malignant TN in patients with HT based on the 6 clinical indicators and radiomic scores from the training group (**Supplementary Table 2**). The diagnostic performance of each model was evaluated using ROC analysis (**Figure 3**). In the training group, the clinical model had an AUC of 0.83 (95% CI: 0.83-0.93). Incorporating the TN score (AUC: 0.90, 95% CI: 0.85-0.94) and TG score (AUC: 0.88, 95% CI: 0.77-0.89) into the model both improved the AUC. The highest AUC (0.94, 95% CI: 0.91-0.98) was achieved when both the TN-USR score and TG-USR score were included in the model. Similar results were obtained when validating the models in the training group. In the Training group, there were significant differences between TN+TG+Clinical model and Clinical model, TN+Clinical model, TG+Clinical model. In the Testing group, only TN+TG+Clinical model exhibited a significant difference when compared to the Clinical model. There were no statistically significant differences observed among the Clinical model, TN+Clinical model, and TG+Clinical model (**Supplementary Table 3**).

**Figure 4:**
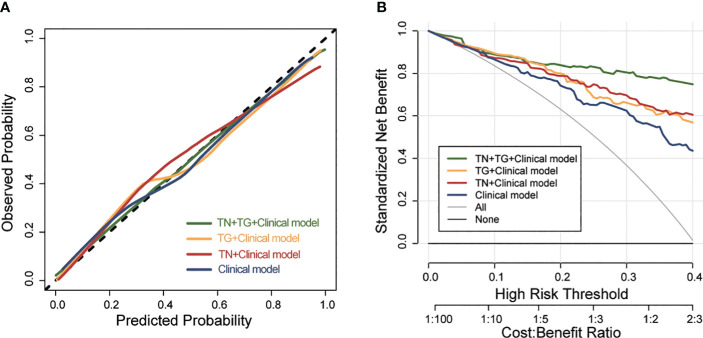
The calibration curve and DCA of different predictive models for predicting TC in training group. **(A)** the calibration curve of different predictive models. **(B)** DCA of different predictive models. DCA, Decision Curve Analysis; TC, thyroid cancer; TN, thyroid nodule; TG, thyroid gland.

Further evaluation of the four models using calibration curves and DCA revealed that the TN+TG+clinical model demonstrated higher diagnostic performance and net benefit (**Figure 4**). Additionally, the TN+TG+Clinical model outperformed the other three models in terms of accuracy (ACC), sensitivity (SEN), specificity (SPE), positive predictive value (PPV), and negative predictive value (NPV) (**Table 3**). Bootstrap internal validation of the model parameters showed that TN+TG USR score, TI-RADS level, boundary, microcalcification, and thyroid function had resampling rates exceeding 50%, indicating their significant predictive value for distinguishing benign and malignant TN in HT patients (**Table 4**).

**Table 3:** Diagnostic performances of models.

Characteristics	ACC, %	SEN, %	SPE, %	PPV, %	NPV, %
Clinical model	0.75(0.74-0.75)	0.82(0.72-0.91)	0.70(0.61-0.79)	0.65(0.54-0.75)	0.85(0.77-0.93)
TN+clinical model	0.82(0.82-0.82)	0.89(0.82-0.97)	0.77(0.69-0.86)	0.73(0.63-0.82)	0.91(0.85-0.98)
TG+clinical model	0.80(0.79-0.80)	0.86(0.78-0.95)	0.75(0.66-0.84)	0.70(0.60-0.80)	0.89(0.82-0.96)
TN+TG+clinical model	0.89(0.89-0.89)	0.86(0.78-0.95)	0.91(0.85-0.97)	0.86(0.78-0.95)	0.91(0.85-0.97)

TN, thyroid nodule; TG, thyroid gland; ACC, indicates accuracy; NPV, negative predictive value; PPV, positive predictive value; SEN, sensitivity; SPE, specificity.

**Table 4:** Bootstrap validation of model.

Characteristics	Risk Ratio	95% CI	Bootstrap percentage (%)
TN+TG USR score	6.90	5.97-7.97	100
TI-RADS level (TR 5 vs TR 4)	8.05	2.21-29.32	100
Echoic (hypo vs iso/hyper)	4.73	0.75-30.01	35
Echoic (marked hypo vs iso/hyper)	9.58	0.70-13.75	32
Boundary (clear vs unclear)	2.69	0.62-11.60	51
Calcification (micro vs NO)	4.56	0.52-40.02	44
Calcification (macro vs NO)	8.80	0.85-90.59	59
Thyroid function (Hyper/hypo-thyroidism vs normal)	8.47	2.16-33.19	95

TN, thyroid nodule; TG, thyroid gland; TI-RADS, Thyroid image reporting and data System; CI, confidence interval.

## 4 Discussion

Nodules are a common manifestation of TC, however, not all TN are malignant, and the majority of them are benign. The benign-malignant discrimination of TN helps in the early detection of TC, guiding treatment decisions, improving patients’ quality of life, and effectively managing healthcare resources. USR can extract a plethora of image features that are not discernible to the naked eye, aiding in the benign-malignant diagnosis of TN. HT is a prevalent autoimmune disease that exhibits a higher coexistence rate with TC. Research suggests that the chronic inflammation associated with HT may contribute to nodule formation. Previous studies on USR features for benign-malignant discrimination of TN have primarily focused on the nodules themselves. However, in patients with HT and TN, both the US features of the nodules and the thyroid gland itself may possess distinct imaging characteristics that can assist in the benign-malignant diagnosis of TN.

USR holds immense promise and advantages in medical research. It not only enables the acquisition of multi-dimensional information but also offers non-invasiveness, real-time imaging, and applicability across various medical fields. Currently, ultrasound technology has been widely applied in the benign and malignant diagnosis of thyroid nodules, including screening models like ACR TI-RADS, European TI-RADS, Chinese TI-RADS, Horvath TI-RADS, and others (11, 29). However, the diagnostic models mentioned above, as reported in many studies, often exhibit a sensitivity and specificity of no more than 80% (29). Radiomic features, capturing tissue and lesion characteristics, can be integrated with histopathological, genomic, or proteomic data to address clinical challenges (30). A multicenter retrospective study revealed that a random forest model based on USR can distinguish endometrial cancer (31). For example, Feng et al. reported that the combined application of radiomics and pathomics could predict the response to neoadjuvant chemoradiotherapy in locally advanced rectal cancer, with high accuracy and specificity (32). Therefore, by advancing and refining the algorithms and techniques of UIR, we can better harness its potential in medical research, enhancing disease diagnosis, treatment, and prognostic evaluation, and promoting personalized medicine.

US is a commonly used diagnostic modality for TC, and USR has been widely studied and explored in the context of TC. US assists in the early diagnosis and screening, malignant risk assessment, preoperative evaluation and surgical guidance, as well as follow-up and prognostic evaluation of TC by assessing the morphological features of TN, internal echogenicity characteristics, and the presence of lymph node metastasis (7, 9, 33, 34). Although there may be subjectivity in the analysis of nodule features, leading to inconsistencies in interpretation among different physicians, extensive research and exploration in the field of USR are addressing this issue (13). Yu et al. identified that the combination of USR features, US features, and clinical factors enables non-invasive preoperative differentiation between thyroid follicular carcinoma and adenoma, potentially reducing unnecessary diagnostic thyroidectomy in patients with benign follicular adenomas (35). Currently, there has been progress in the application of USR in TC and TN, but challenges remain regarding the accuracy of malignant risk assessment, nodule classification and boundary delineation, establishment and sharing of datasets, and clinical validation (13). Through further research and efforts, we can gradually overcome these challenges. Additionally, our study can expand the application of USR in patients with TN associated with HT, thereby advancing the clinical application of USR in thyroid diseases.

Due to its high sensitivity, non-ionizing radiation, ease operating, and rapid diagnosis, US is the preferred method for screening of TN. In recent years, new US techniques such as contrast-enhanced US and US elastography have greatly improved the diagnostic accuracy of TN (36). For example, Liang et al. found that the diagnostic performance of USR score derived from US image were not worse than the ACR TI-RADS (37). However, diagnosing TC in HT patients can be challenging, as HT itself causes inflammation and nodular formation in the thyroid tissue, making differentiation from malignant lesions on US images difficult (38, 39). Several studies have demonstrated the significant predictive value of US features and USR in HT patients with TC. Feng et al. found that US grayscale ratio was independently associated with central compartment lymph node metastasis in patients with HT (40), while Jin et al. developed a prediction model for central compartment lymph node metastasis in patients with HT based on USR (27). Clearly, USR features play an important role in distinguishing the benign and malignant nature of TN in HT patients, and further exploration is needed.

Our study has shown that USR features of glands combined nodules in patients with HT can improve the accuracy of benign-malignant discrimination of TN. This may be attributed to the close association between certain USR features and the pathological processes of TC in the presence of HT. Firstly, the immunological characteristics of HT, such as the production of autoantibodies, T-cell mediated immune responses, and immune tolerance abnormalities, might be reflected by USR features (21). Previous studies have demonstrated that radiomic features of immune cells, particularly tumor-infiltrating lymphocytes, can predict the prognosis of tumor treatment (41–43). Furthermore, certain USR features have been found to correlate with the presence of malignant gene mutations in TC. Wang et al. reported that a radiomics model based on grayscale and elastography ultrasound had good predictive value for the BRAF-V600E gene mutation in patients with TC (44). Therefore, in future research, integrating radiomics with pathology, genetics, and immunology would greatly enhance our understanding of the correlation between radiomics features and the benign-malignant nature of TC in the presence of HT.

The study has several limitations. Firstly, it is a small-sample retrospective study, and selection bias is inevitable. To validate the research findings and provide stronger evidence, standardized protocols and larger prospective studies are needed. Secondly, the focus on collecting TN images in clinical imaging may lead to inconsistency in US images of the TG affected by HT, which could impact the extraction of radiomic features for the TG in HT. Lastly, the correlation between TC and HT in terms of disease occurrence is still a matter of debate, and it remains unknown whether the radiomic features can be linked to the pathological process of TC induced by HT. In conclusion, further clinical and mechanistic studies are still needed in this research direction to guide the clinical diagnosis of TC.

## 5 Conclusion

Our study provides compelling evidence that integrating the USR features of TN with the specific features of the TG in patients with HT significantly enhances the differentiation between benign and malignant TN. The TN+TG+clinical model exhibited superior performance compared to other models, demonstrating higher accuracy and net benefit. These findings underscore the critical importance of considering the entire TG, alongside TN characteristics, in the evaluation of TN in HT patients. This comprehensive approach holds valuable implications for clinical decision-making, facilitating more accurate diagnosis and management strategies in this specific patient population. Further research and validation are warranted to confirm the robustness and generalizability of our findings.

## Data availability statement

The raw data supporting the conclusions of this article will be made available by the authors, without undue reservation.

## Ethics statement

The studies were conducted in accordance with the local legislation and institutional requirements. The participants provided their written informed consent to participate in this study.

## Author contributions

MF: Conceptualization, Data curation, Investigation, Software, Writing – original draft. ML: Data curation, Formal Analysis, Methodology, Project administration, Resources, Software, Writing – original draft. XC: Data curation, Formal Analysis, Investigation, Methodology, Project administration, Supervision, Writing – original draft. HC: Methodology, Project administration, Resources, Validation, Writing – original draft. XD: Formal Analysis, Methodology, Resources, Software, Supervision, Writing – original draft. HY: Supervision, Validation, Writing – review & editing. LG: Funding acquisition, Resources, Supervision, Validation, Visualization, Writing – review & editing.
