# Supplementary material for: Radiomics-based ultrasound models for thyroid nodule differentiation in Hashimoto’s thyroiditis
Source: Front Endocrinol (Lausanne). 2023 Oct 23;14:1267886. doi: 10.3389/fendo.2023.1267886 (PMC10627229; doi:10.3389/fendo.2023.1267886)
Supplement: Supplementary file 1 [file Table_1.docx]

**Supplementary table 1. Univariate analysis for TN status in the training and the test datasets.**

| **Characteristics** | **Training group** | |  | **Testing group** | |
| --- | --- | --- | --- | --- | --- |
|  | **OR (95%CI)** | **p value** |  | **OR (95%CI)** | **p value** |
| Age, continuous | 1.00 (0.97-1.03) | 0.95 |  | 0.99(0.95-1.04) | 0.73 |
| Gender (male vs female) | 1.02(0.39-2.65) | 0.97 |  | 0.27(0.02-3.13) | 0.29 |
| TI-RADS level (TR 5 vs 4) | 16.5(7.29-37.35) | <0.001 |  | 12.5(3.53-44.3) | <0.001 |
| Echoic type (hypo vs iso/hyper echoic) | 5.51(1.82-16.7) | <0.001 |  | 6.50(0.77-54.85) | 0.09 |
| Echoic type (marked hypo vs iso/hyper echoic) | 52.5(8.57-321.46) | <0.001 |  | 117(6.44-212.74) | <0.001 |
| Aspect ratio (>1 vs ≤1) | 5.10(2.49-10.45) | <0.001 |  | 4.00(1.32-12.11) | 0.01 |
| Boundary (unclear vs clear) | 4.28(1.95-9.38) | <0.001 |  | 3.14(0.99-9.99) | 0.05 |
| Margin  (ill-defined vs well-defined) | 0.84(0.44-1.59) | 0.59 |  | 0.55(0.20-1.52) | 0.25 |
| Calcification (macro calcification vs NO) | 7.60(2.04-28.38) | <0.001 |  | 3.20(0.67-15.38) | 0.15 |
| Calcification (micro calcification vs NO) | 9.11(2.56-32.35) | <0.001 |  | 2.60(0.61-11.03) | 0.19 |
| Vascularization (low/median vs NO) | 2.25(0.68-7.47) | 0.19 |  | 0.28(0.02-3.32) | 0.31 |
| Vascularization (high vs NO) | 2.04(0.57-7.27) | 0.27 |  | 0.25(0.02-3.25) | 0.29 |
| Thyroid function(Hyper/hypo-thyroidism vs normal) | 3.47(1.73-6.99) | <0.001 |  | 3.57(1.13-11.25) | 0.03 |
| Thyroid function index, continuous |  |  |  |  |  |
| TSH | 0.99(0.95-1.02) | 0.44 |  | 1.00(0.95-1.05) | 0.94 |
| PTH | 0.99(0.97-1.01) | 0.38 |  | 0.99(0.96-1.02) | 0.53 |
| TPO | 1.00 (1.00-1.00) | 0.49 |  | 1.00(1.00-1.00) | 0.81 |
| Thyroid globulin | 1.00(1.00-1.01) | 0.29 |  | 1.00(1.00-1.01) | 0.25 |
| anti-TG | 1.00 (1.00-1.00) | 0.03 |  | 1.00(1.00-1.01) | 0.05 |
| calcitonin | 0.95(0.87-1.03) | 0.19 |  | 0.88(0.75-1.02) | 0.09 |
| FT3 | 1.07(0.94-1.23) | 0.31 |  | 1.05(0.94-1.17) | 0.4 |
| FT4 | 1.00(1.00-1.01) | 0.67 |  | 1.00(1.00-1.01) | 0.55 |
| TT3 | 1.00 (1.00-1.00) | 0.91 |  | 1.00(1.00-1.01) | 0.79 |
| TT4 | 1.01(1.00-1.02) | 0.10 |  | 1.01(0.99-1.02) | 0.33 |
| USR score, continuous |  |  |  |  |  |
| TG | 9.11(1.61-94.14) | <0.001 |  | 6.28(2.11-18.69) | <0.001 |
| TN | 16.37(2.43-95.38) | <0.001 |  | 18.72(4.08-73.09) | <0.001 |
| TN+TG | 33.01(64.36-175.06) | <0.001 |  | 20.8(7.38-234.02) | <0.001 |

OR:odds ratio; TSH: thyroid stimulating hormone; PTH: parathyroid hormone; TPO: thyroid peroxidase;

anti-TG: anti-thyroid globulin; FT3, free hypothyroidism; FT4, free neurotoxin; TT3, total hypothyroidism;

TT4, total neurotoxin; USR: ultrasound radiomics; TG: thyroid gland;TN: thyroid nodule.
