# Supplementary material for: Radiomics-based ultrasound models for thyroid nodule differentiation in Hashimoto’s thyroiditis
Source: Front Endocrinol (Lausanne). 2023 Oct 23;14:1267886. doi: 10.3389/fendo.2023.1267886 (PMC10627229; doi:10.3389/fendo.2023.1267886)
Supplement: Supplementary file 2 [file Table_2.docx]

**Supplementary table 2. Multivariate analysis for TN status in the training and testing group.**

| **Characteristics** | **Clinical** | |  | **TG+Clinical** | |  | **TN+Clinical** | |  | **TN+TG+Clinical model:** | |
| --- | --- | --- | --- | --- | --- | --- | --- | --- | --- | --- | --- |
|  | **OR (95%CI)** | **p value** |  | **OR (95%CI)** | **p value** |  | **OR (95%CI)** | **p value** |  | **OR (95%CI)** | **p value** |
| **Training group** |  |  |  |  |  |  |  |  |  |  |  |
| TI-RADS level (TR 5 vs 4) | 6.13(2.09-18.01) | <0.001 |  | 4.68(1.39-15.73) | 0.01 |  | 4.98(1.56-15.89) | 0.01 |  | 5.51(1.2-25.4) | 0.03 |
| Echoic type (hypo vs iso/hyper echoic) | 4.43(1.15-17.01) | 0.03 |  | 2.7(0.6-12.17) | 0.19 |  | 6.68(1.47-30.28) | 0.01 |  | 4.73(0.77-29.02) | 0.09 |
| Echoic type (marked hypo vs iso/hyper echoic) | 11.8(1.53-91.14) | 0.02 |  | 12.87(1.3-127.03) | 0.03 |  | 18.35(1.96-171.47) | 0.01 |  | 11.51(0.76-174.32) | 0.08 |
| Aspect ratio (>1 vs ≤1) | 2.27(0.86-6.02) | 0.1 |  | 3.54(1.1-11.35) | 0.03 |  | 2.31(0.78-6.81) | 0.13 |  | 1.85(0.47-7.3) | 0.38 |
| Boundary (unclear vs clear) | 1.87(0.66-5.28) | 0.24 |  | 1.81(0.55-5.91) | 0.33 |  | 2.27(0.76-6.81) | 0.14 |  | 2.58(0.6-11.02) | 0.2 |
| Calcification (macro calcification vs NO) | 4.98(1.02-24.32) | 0.05 |  | 4.86(0.77-30.62) | 0.09 |  | 5.57(1-31.04) | 0.05 |  | 8.68(0.84-90.08) | 0.07 |
| Calcification (micro calcification vs NO) | 3.25(0.7-15.03) | 0.13 |  | 2.8(0.47-16.76) | 0.26 |  | 4.83(0.92-25.41) | 0.06 |  | 4.8(0.54-42.71) | 0.16 |
| Thyroid function(Hyper/hypo-thyroidism vs normal) | 4.33(1.64-11.4) | <0.001 |  | 4.2(1.4-12.61) | 0.01 |  | 5.68(1.92-16.81) | <0.001 |  | 7.93(2.03-30.91) | <0.001 |
| TG-USR score | - | - |  | 16.95(1.78-175.04) | <0.001 |  | - | - |  | - | - |
| TN-USR score | - | - |  | - | - |  | 54.13(1.48-166.29) | <0.001 |  | - | - |
| TN+TG-USR score | - | - |  | - | - |  | - | - |  | 62.42(54.83-717.41) | <0.001 |
| **Testing group** |  |  |  |  |  |  |  |  |  |  |  |
| TI-RADS level (TR 5 vs 4) | 4.54(0.84-24.56) | 0.08 |  | 3.56(0.55-22.84) | 0.18 |  | 11.99(1.2-120.2) | 0.03 |  | 20.01(1.23-324.44) | 0.04 |
| Echoic type (hypo vs iso/hyper echoic) | 4.27(0.42-43.88) | 0.22 |  | 5.94(0.47-75.4) | 0.17 |  | 0.89(0.06-12.89) | 0.93 |  | 2.05(0.11-38.33) | 0.63 |
| Echoic type (marked hypo vs iso/hyper echoic) | 23.47(1.02-540.78) | 0.05 |  | 38.16(1.24-117.11) | 0.04 |  | 2.24(0.06-85.72) | 0.67 |  | 2.93(0.08-104.61) | 0.56 |
| Aspect ratio (>1 vs ≤1) | 1.57(0.33-7.58) | 0.57 |  | 1.83(0.3-11.2) | 0.51 |  | 1.92(0.28-13.36) | 0.51 |  | 0.96(0.11-8.35) | 0.97 |
| Boundary (unclear vs clear) | 1.55(0.35-6.89) | 0.57 |  | 1.74(0.36-8.36) | 0.49 |  | 1.01(0.17-6.18) | 0.99 |  | 1.10(0.15-7.97) | 0.93 |
| Calcification (macro calcification vs NO) | 1.63(0.22-12.21) | 0.63 |  | 1.91(0.21-17.27) | 0.57 |  | 1.56(0.12-21.2) | 0.74 |  | 3.58(0.16-82.5) | 0.42 |
| Calcification (micro calcification vs NO) | 1.22(0.21-7.21) | 0.83 |  | 1.5(0.22-10.24) | 0.68 |  | 0.91(0.08-10) | 0.94 |  | 2.65(0.2-34.65) | 0.46 |
| Thyroid function(Hyper/hypo-thyroidism vs normal) | 3.29(0.72-15.13) | 0.13 |  | 6.07(0.98-37.66) | 0.05 |  | 2.41(0.43-13.47) | 0.32 |  | 5.12(1.81-32.18) | 0.01 |
| USR score, continuous | | | | | | | | | | | |
| TG-USR score | - | - |  | 34.96(1.97-61.76) | 0.03 |  | - | - |  | - | - |
| TN-USR score | - | - |  | - | - |  | 42.92(1.8-95.32) | <0.001 |  | - | - |
| TN+TG-USR score | - | - |  | - | - |  | - | - |  | 32.84(14.19-360.81) | <0.001 |

OR:odds ratio; USR: ultrasound radiomics; TG: thyroid gland;TN: thyroid nodule; TI-RADS: thyroid image reporting and data system;
