# Supplementary material for: Radiomics-based ultrasound models for thyroid nodule differentiation in Hashimoto’s thyroiditis
Source: Front Endocrinol (Lausanne). 2023 Oct 23;14:1267886. doi: 10.3389/fendo.2023.1267886 (PMC10627229; doi:10.3389/fendo.2023.1267886)
Supplement: Supplementary file 3 [file Table_3.docx]

| Model comparison | Training group | Testing group |
| --- | --- | --- |
| Clinical vs TN+Clinical | 0.030 | 0.115 |
| Clinical vs TG+Clinical | 0.041 | 0.139 |
| Clinical vs TN+TG+Clinical | <0.001 | 0.032 |
| TN+Clinical vs TG+Clinical | 0.691 | 0.651 |
| TN+Clinical vs TN+TG+Clinical | 0.015 | 0.441 |
| TG+Clinical vs TN+TG+Clinical | 0.006 | 0.250 |

**Supplementary table 3. p-values for comparing ROC curves among different models.**

TN, thyroid nodule; TG, thyroid gland.
